# Leptomeningeal and intramedullary metastases of glioblastoma multiforme in a patient reoperated during adjuvant radiochemotherapy

**DOI:** 10.1186/1477-7819-11-55

**Published:** 2013-03-05

**Authors:** Josip Joachim Grah, Darko Katalinic, Ranka Stern-Padovan, Josip Paladino, Fedor Santek, Antonio Juretic, Kamelija Zarkovic, Stjepko Plestina, Marijana Supe

**Affiliations:** 1 Department of Oncology, University Hospital Centre (KBC Zagreb), University of Zagreb School of Medicine, Kispaticeva 12, Zagreb HR-10000, Croatia; 2 Department of Diagnostic and Interventional Radiology, University Hospital Centre (KBC Zagreb), University of Zagreb School of Medicine, Zagreb, Croatia; 3 Department of Neurosurgery, University Hospital Centre (KBC Zagreb), University of Zagreb School of Medicine, Zagreb, Croatia; 4 Department of Pathology and Cytology, University Hospital Centre (KBC Zagreb), University of Zagreb School of Medicine, Zagreb, Croatia

**Keywords:** Glioblastoma multiforme, Leptomeningeal and intramedullary metastases, Radiochemotherapy

## Abstract

Despite huge advances in medicine, glioblastoma multiforme (GBM) remains a highly lethal, fast-growing tumour that cannot be cured by currently available therapies. However, extracranial and extraneural dissemination of GBM is extremely rare, but is being recognised in different imaging studies. To date, the cause of the GBM metastatic spread still remains under discussion. It probably develops at the time of intracranial progression following a surgical procedure. According to other hypothesis, the metastases are a consequence of spontaneous tumour transdural extension or haematogenous dissemination. We present a case of a 59-year-old woman with symptomatic leptomeningeal and intramedullary metastases of GBM who has been previously surgically treated with primary subtotal resection and underwent a repeated surgery during adjuvant radiotherapy and chemotherapy with temozolomide. Today, the main goal of surgery and chemoradiotherapy is to prevent neurologic deterioration and improve health-related quality of life. With this paper, we want to present this rare entity and emphasise the importance of a multidisciplinary approach, a key function in the management of brain tumour patients. The prognosis is still very poor although prolongation of survival can be obtained. Finally, although rare, our case strongly suggests that clinicians should be familiar with the possibility of the extracranial spread of GBM because as treatment improvements provide better control of the primary tumour and improving survival, metastatic disease will be increasingly encountered.

## Background

Glioblastoma multiforme (GBM) is the most common primary malignancy of the central nervous system (CNS) in adults. Macroscopically evident and symptomatic spinal metastases occur rarely, in up to 2% to 5% of patients [1,2]. Spread or dissemination within the neuraxis [3] is commoner than spread to other areas like the vertebral body and peritoneum which have also been reported [4,5]. We present a rare case of symptomatic spinal leptomeningeal and intramedullary metastases of GBM in a patient who has been previously surgically treated with primary subtotal resection and underwent a repeated surgery during adjuvant radiotherapy and chemotherapy with temozolomide.

## Case presentation

**Figure 1:**
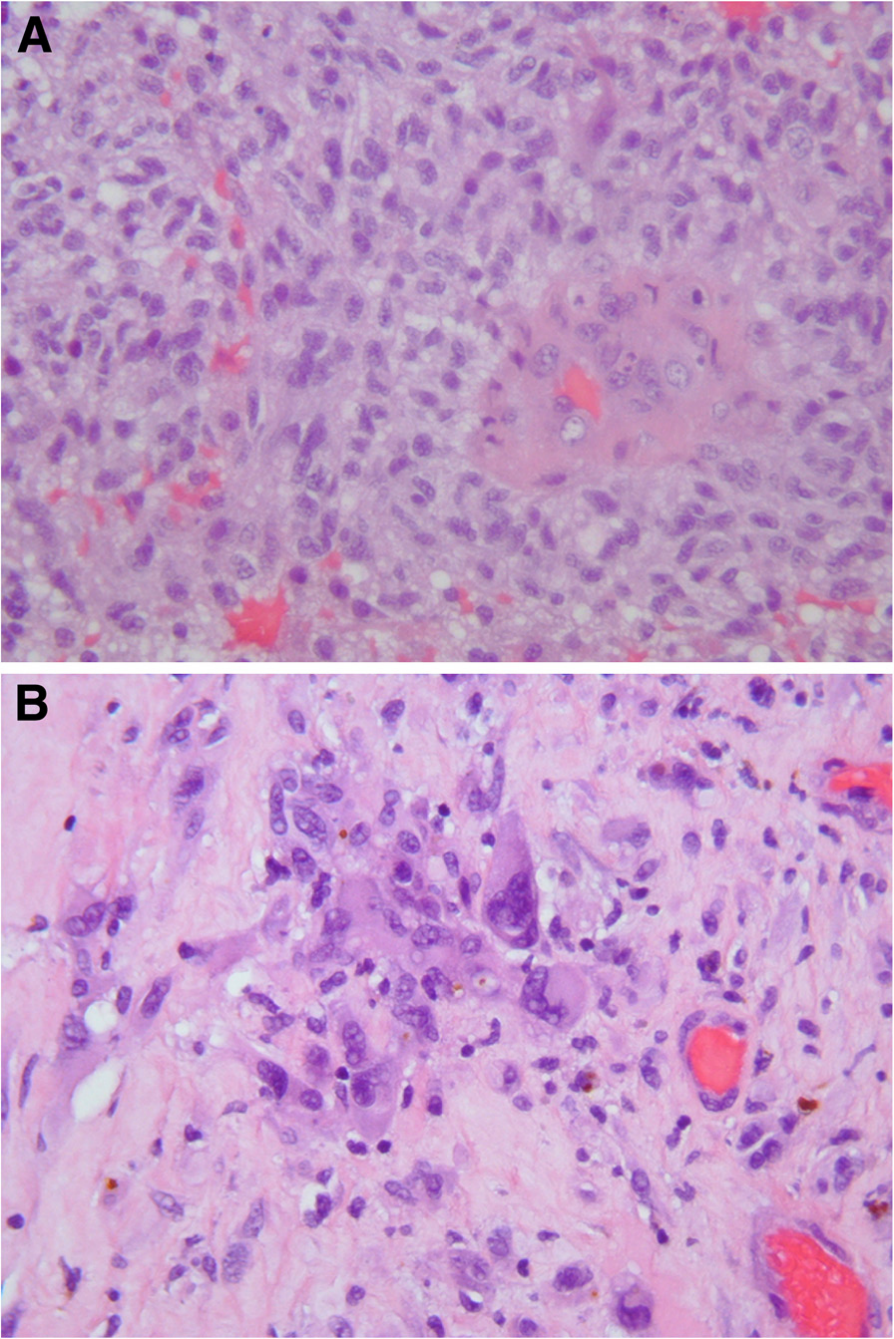
**Histopathological evaluation.** Hematoxylin and eosin (HE) histologic analysis revealed a highly cellular tumour tissue composed of pleomorphic astroglial cells with hyperchromatic nuclei, mitosis and glomeruloid vascular proliferation, which are a classic histological features in glioblastoma multiforme (**A**, high-power photomicrograph, original magnification, ×400). After 48Gy chemoradiotherapy, previously treated glioblastoma shows heterogeneos composition with area of coagulative necrosis and hyalinized blood vesels. Nuclear pleomorphism of tumour cells without mitosis were noted (**B**, high-power photomicrograph, original magnification, ×400).

**Figure 2:**
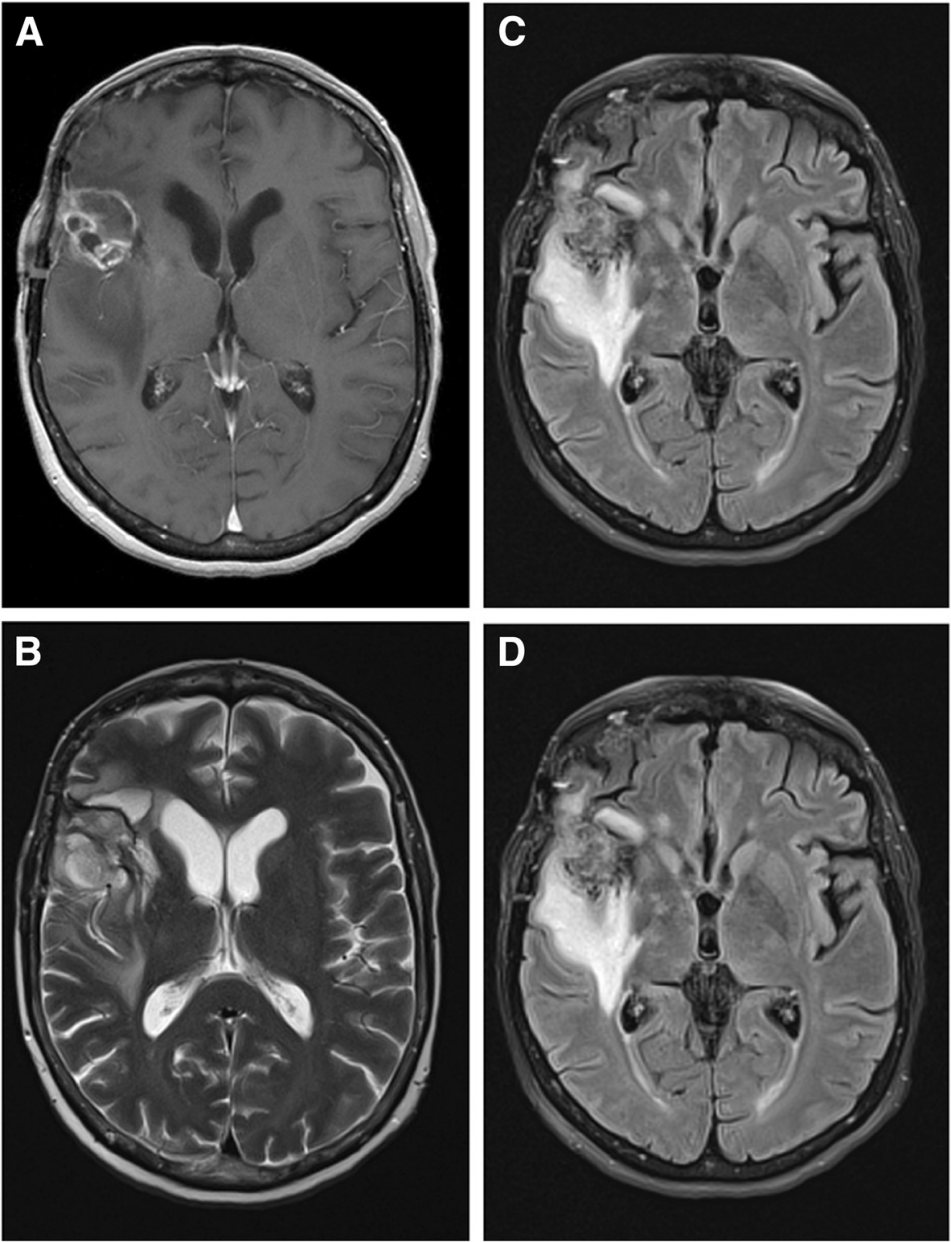
**Radiological evaluation of the brain.** T1-weighted axial gadolinium-enhanced magnetic resonance image demonstrates an enhancing tumour of the right frontal lobe (**A**). T2-weighted image demonstrates the same lesion as in the previous image, with notable tissue edema (**B**). This finding is consistent with a high-grade glioblastoma. On fast fluid-attenuated inversion-recovery (FLAIR) MRI scan a zone of edema is identified around the tumour demonstrating increased signal intensity (**C**, **D**).

**Figure 3:**
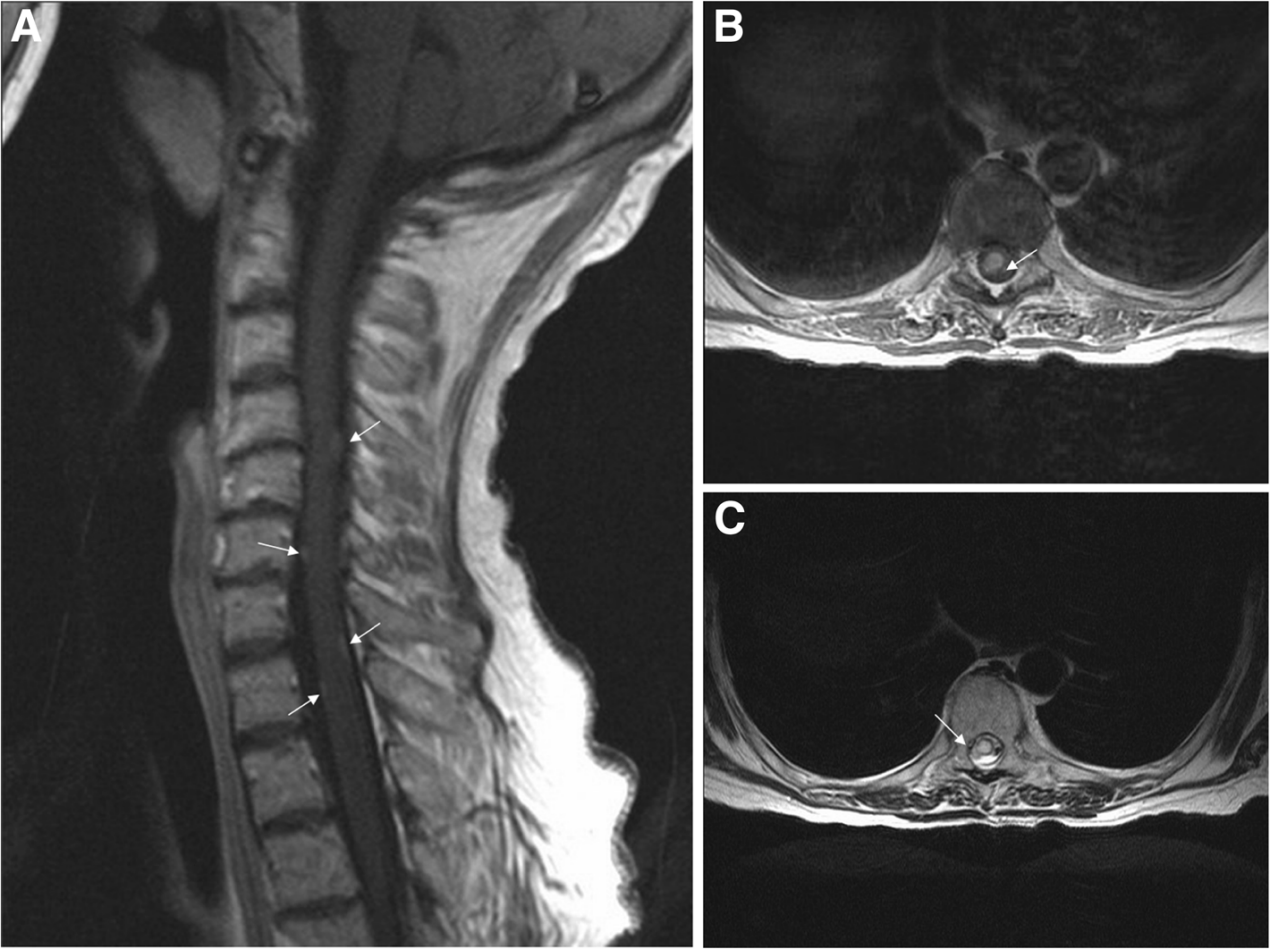
**Radiological evaluation of the spinal cord.** MRI of the cervical spine demonstrated an enhancing cervical leptomeningeal metastases (arrows) at the level C3 to C7. (**A**, T1-weighted image). Additionally, at the T8 to T10 level, MRI also revealed intramedullary metastases with extensive contrast enhancement, central necrosis and vasogenic edema (arrows) (**B**, T1-weighted image) and **C** (T2-weighted image).

In June 2011, a 59-year-old white female presented with headaches and right facial palsy of the central type. Magnetic resonance imaging (MRI) of the brain showed a lesion in the right frontal area. A right frontotemporal craniotomy for the mass resection was performed. Intraoperatively, a quite irregular and partially necrotic tumour mass was noted. The pathohistology analysis demonstrated marked nuclear pleomorphism, scattered mitoses and vascular endothelial proliferation with necrosis, consistent with the diagnosis of GBM (World Health Organization (WHO) grade IV) (Figure 1A). The patient had a good Karnofsky performance status (KPS >70) without any aberration of cognitive functions. Six weeks after surgery, she was treated with standard adjuvant radiotherapy (three-dimensional conformal external beam radiotherapy (3D-CRT)) with concurrent chemotherapy (temozolomide 75 mg/m^2^ per day) with regular daily administration of dexamethason 12 mg i.m. During chemoradiotherapy (after 24 of 30 fractions), she underwent an MRI examination due to exacerbation of the neurological signs and tumour progression was noted (Figure 2). A right frontotemporal recraniotomy was performed with preoperative administration of Gliolan. Using fluorescent light, a tumour that encompassed arteria cerebri media and its branches in fissura Sylvii were noted and a subtotal resection was performed. The mass was firm and fixed with blood vessels. A histopathological examination confirmed GBM (Figure 1B). A decision to re-resect the tumour was made by the neurosurgeon in consultation with other members of the team, taking into consideration the good performance status of the patient. Adjuvant radiotherapy and chemotherapy (temozolomide 75 mg/m^2^ per day) continued with 16 Gy in eight fractions (total dose of 60 Gy in 30 fractions). Sequential chemotherapy with temozolomide (150 to 200 mg/m^2^ for 5 days during each 28-day cycle) was discontinued after two cycles because the patient complained of back pain and proximal weakness in both lower limbs with paraesthesia. MRI of the cervical spine demonstrated enhancing cervical leptomeningeal metastases on a level of C3 to C7. Additionally, on a T8 to T10 level, MRI also revealed intramedullary metastases with extensive contrast enhancement, central necrosis and vasogenic oedema (Figure 3). A follow-up MRI of the brain showed no significant intracranial changes. Palliative radiotherapy to C3 to C6 and T8 to T10 with a total dose of 30 Gy in 10 fractions was delivered because at that point, the patient was still in good general condition. The palliative effects in terms of pain relief lasted until the patient’s death 1 month after the diagnosis of spinal metastases. The patient probably died as a result of extracranial or intracranial disease progression (presumably both). No autopsy was performed as requested by the family.

## Discussion

Gliomas comprise a heterogeneous group of tumours that differ in location within the CNS, in age and sex distribution, in morphological features, in tendency to progression, and in response to surgical and oncological treatment. GBM remains the most aggressive of gliomas, a collection of tumours developing from glial tissue or their precursors, accounting for 12% to 15% of all intracranial neoplasms [2]. First described by Rudolph Virchow in 1863, it represents the most common tumour of the cerebral hemispheres, usually occurring between the ages of 40 and 60 years old. Genetic alterations such as point mutations, loss of heterozygosity or excessive activation of a particular genes are all reported in GBMs, but intrinsic risk factors are currently unknown [6]. Evidence of association with occupational risk factors, head injury or exposure to electromagnetic fields is inconclusive. The incidence in Europe and North America is two to three cases/100,000 per year [7]. However, much less commonly, GBM can affect the brainstem (especially in children) and the spinal cord [8,9]. Clinically, gliomas are divided into four grades; the most aggressive of these, grade IV or GBM, is also the most common in humans. These tumours may develop from lower-grade astrocytomas (WHO grade II) or anaplastic astrocytomas (WHO grade III) (secondary GBM), but more frequently they manifest *de novo*, without any evidence of less malignant precursors (primary GBM) [10,11]. They all typically contain both neoplastic and stromal cells, which contribute to their heterogeneity and variable outcome. Molecular studies and gene profiling potentially allow for better classification of these tumours and their division into different prognostic groups.

With the exception of brainstem gliomas, GBM has the worst prognosis of any CNS malignancy, despite multimodality treatment. The current standard of care for glioblastoma is surgical resection of as much of the tumour as is safe and possible, followed by postoperative radiation therapy (focal irradiation at a dose of 2 Gy/fraction given once daily 5 days per week over a period of 6 weeks, for a total dose of 60 Gy) and chemotherapy (temozolomide at a dose of 75 mg/m^2^/day, given 7 days per week from the first day of radiotherapy up to the last day of radiotherapy, but for no longer than 49 days). After a 4-week break, patients were then to receive up to six cycles of temozolomide according to the standard 5-day schedule every 28 days. [12]. To date, systemic delivery of different chemotherapeutic agents to GBM has had limited efficiency with significant side effects including haematological toxicity, specifically thrombocytopenia and neutropenia. Even under the best of circumstances, where all of the tumour seen on MRI scan can be surgically removed and patients are fully treated with radiochemotherapy, over 75% of patients die within 18 months and essentially none attain long-term survival [13,14]. However, some progress is being made in the field of molecular neuro-oncology, which involves an understanding of the mutations and expression of genes that have been implicated with GMB pathophysiology [15-23]. Such procedures may be helpful to identify the tumour and they also could facilitate therapy as potential vectors of treatment [24,25]. The main objective is not only to generate more new drugs; it is also to find different tools to assault specific types of different brain tumours. Finally, a genetic profile could be the deciding factor in the diagnosis and treatment for each patient individually, while best meeting cost/benefit issues.

Notwithstanding all this, the treatment of GBM still remains one of the most disappointing challenges in modern oncology. A surgical cure for these brain tumours is virtually impossible to provide. The clinical course is determined by the biology of the tumour and response to surgery, radiation and chemotherapy [26]. In our patient, the treatment was multimodal and multidisciplinary, based on cytoreductive surgery followed by chemotherapy (temozolomide) and radiotherapy. As we know, surgical intervention and radical resection are essential in the initial treatment while the extent of surgery can affect overall patient survival [27-30]. When faced with evidence of recurrent or metastatic GBM, surgical intervention requires identification of goals and a clear consideration of overall prognosis, including all treatment side effects. In patients with good KPS (>70) and without significant contraindications, surgery can improve neurological status and reduce intracranial pressure. Repeated resection should only be considered in patients with high preoperative KPS (>70) or in those whose symptoms are secondary to mass effect from superficial regions and whose lesions are in a favourable brain location [30]. Radiological follow-up should include brain/spinal MRI every 4 to 6 months after the cranial removal [31]. However, the efficacy of repeated resection alone in cases of recurrent GBM remains controversial due to a lack of randomized clinical trials. Some studies showed that the median survival time after resection was 14 to 50 weeks [32-34].

As mentioned earlier, spinal or systemic GBM metastases are very rare [1,2]. This could be because of patient death before clinically detectable spreading or impediments to systemic egress. Spinal and dural metastases should be commonly suspected in all patients with a history of intracranial GBM who complain about signs and symptoms not explained by the primary lesion. The clinical diagnosis of these conditions may be difficult, but is possible with careful neurological examination directed at radicular signs. Spinal MRI with contrast enhancement has a leading diagnostic role in patients with serious spinal pathology and should be considered in the presence of any clinical signs of spinal involvement [35,36]. To date, the cause of the tumour spread still remains under discussion. It probably develops at the time of intracranial progression following a surgical procedure. According to other hypothesis, spinal metastases most likely occur as a result of cellular spread in the subarachnoidal space or haematogenous dissemination [2]. Any new onset of axial back pain or neurological deficit of the extremities in patients with prior diagnosis of GBM should indicate suspected spinal metastasis, however rarely they may occur.

The prognosis in cases of leptomeningeal metastasis is very poor, regardless of the treatment modality used. The survival of patients is approximately 2 to 3 months [37,38]. Radiotherapy is the most common treatment modality with 3 to 4 Gy per fraction to a total dose of up to 40 Gy [39]. Radiotherapy may provide relief of pain and some improvement in neurological function, but no survival benefits. Systemic chemotherapy or intraventricular chemotherapy in combination with radiotherapy has also not improved overall survival [40]. Surgery may enhance the risk of drop metastases, although these have also been reported in patients who have never had a surgical procedure [41].

## Conclusion

Although merely a theoretical possibility in most cases, reoperation of GBM during adjuvant radiochemotherapy was still feasible in our patient. Today, the main objective of chemoradiotherapy in patients with metastatic GBM is to prevent neurologic deterioration and improve health-related quality of life [26]. Researchers continue to study the common characteristics of GBM and how personalized and targeted treatments may be optimally used. With this paper, we not only aim to present this rare entity, but also to emphasise the importance of a multidisciplinary approach, a key function in the management of tumour patients. Additionally, clinicians should be familiar with the possibility of extracranial spreading of GBM because as treatment improvements provide better control of the primary tumour and improving survival, metastatic disease will be increasingly encountered.

## Consent

Written informed consent was obtained from the patient to the publication of this case report and any accompanying images. A copy of the written consent is available for review by the Editor-in-Chief of this journal.

## Abbreviations

3D-CRT: Three-dimensional conformal external beam radiotherapy; CNS: Central nervous system; GBM: Glioblastoma multiforme; Gy: Gray; KPS: Karnofsky performance status; MRI: Magnetic resonance imaging; WHO: World Health Organization.

## Competing interests

Each co-author certifies that she/he has no commercial interest that might constitute a conflict of interest in connection with the submitted article.

## Authors’ contribution

JJG, FS and RSP were responsible for the writing. JP, MS and KZ participated in data collection. DK, AJ and SP participated in literature searching and helped draft the manuscript. All authors have read and approved the final manuscript.
